# Antimicrobial Peptides as Anticancer Agents: Functional Properties and Biological Activities

**DOI:** 10.3390/molecules25122850

**Published:** 2020-06-19

**Authors:** Anna Lucia Tornesello, Antonella Borrelli, Luigi Buonaguro, Franco Maria Buonaguro, Maria Lina Tornesello

**Affiliations:** 1 Molecular Biology and Viral Oncology Unit, Istituto Nazionale Tumori IRCCS “Fondazione G. Pascale”, 80131 Napoli, Italy; f.buonaguro@istitutotumori.na.it (F.M.B.); m.tornesello@istitutotumori.na.it (M.L.T.); 2 Innovative Immunological Models, Istituto Nazionale Tumori IRCCS “Fondazione G. Pascale”, 80131 Napoli, Italy; l.buonaguro@istitutotumori.na.it

**Keywords:** antimicrobial peptides, AMPs, cancer, anticancer peptides, ACPs

## Abstract

Antimicrobial peptides (AMPs), or host defense peptides, are small cationic or amphipathic molecules produced by prokaryotic and eukaryotic organisms that play a key role in the innate immune defense against viruses, bacteria and fungi. AMPs have either antimicrobial or anticancer activities. Indeed, cationic AMPs are able to disrupt microbial cell membranes by interacting with negatively charged phospholipids. Moreover, several peptides are capable to trigger cytotoxicity of human cancer cells by binding to negatively charged phosphatidylserine moieties which are selectively exposed on the outer surface of cancer cell plasma membranes. In addition, some AMPs, such as LTX-315, have shown to induce release of tumor antigens and potent damage associated molecular patterns by causing alterations in the intracellular organelles of cancer cells. Given the recognized medical need of novel anticancer drugs, AMPs could represent a potential source of effective therapeutic agents, either alone or in combination with other small molecules, in oncology. In this review we summarize and describe the properties and the mode of action of AMPs as well as the strategies to increase their selectivity toward specific cancer cells.

## 1. Introduction

Antimicrobial peptides (AMPs) are short amino acid sequences produced by all organisms, from bacteria to mammals, which act as primary defense against a broad spectrum of pathogens [1].The first recognized AMP was lysozyme, discovered by Alexander Fleming in 1922, which was detected in vegetables as well as animal tissues and secretions and shown to possess bacteriolytic activity [2]. In the following decade, Dubos extracted gramicidin from a soil *Bacillus* strain and demonstrated that it was able to protect mice from pneumococci infection and to be effective for topical treatment of wounds and ulcers [3,4,5,6].

In mammals, leukin and phagocytin were the first animal-derived AMPs that were isolated from rabbit leukocytes in 1956 [7]. Few years later, “bactericidal basic proteins” were demonstrated to accumulate in the lysosomes of human leukocytes [8]. In 1985, the Lehrer group extracted three small peptides (HNP-1, HNP-2, and HNP-3) from human neutrophils, referred to as “defensins”, that were demonstrated to kill *Staphylococcus aureus*, *Pseudomonas aeruginosa*, and *Escherichia coli* [9,10,11]. During the past decades, a large number of natural and synthetic AMPs were characterized and public databases were established, such as APD3, CAMP and DRAMP 2.0, which include the list of natural AMPs, the classification of AMP families and AMP clinical applications, respectively [12,13,14]. Databases of specific peptides have been also developed such as the Defensins Knowledgebase, the antiviral AVPdb, the antiparasitic ParaPep and the anticancer CancerPPD [15,16,17,18].

AMPs are short peptides, mostly less than 100 amino acids long, that in general do not share conserved motifs but are characterized by net positive charges and high proportions of hydrophobic residues [19]. As a result, they tend to establish non-specific interactions with negatively charged phospholipids, such as the phosphatidylglycerol, that are particularly abundant in the microbial membranes causing increased permeability, leakage of cytoplasmic components and cell death [20,21,22,23]. The effective bacteriolytic activity of AMPs is regulated by chemical and physical properties including hydrophobicity, electrostatic and hydrophobic forces, conformational transitions and self-assembly equilibria that finally modulate the membrane-binding affinity [20]. The antiviral activity of several AMPs occurs both at virus entry into the cells as well as at other stages of virus infection, particularly affecting viral trafficking within infected cells [24,25]. Among these defensins have shown to act against several viruses, such as human immunodeficiency virus (HIV), respiratory syncytial virus (RSV), herpes simplex virus (HSV), papillomavirus (HPV) and severe acute respiratory syndrome coronavirus (SARSC) [26,27].

Several AMPs have shown to selectively target human tumor cells through their ability to bind the phospholipid phosphatidylserines (PS) that in cancer cells are mainly localized in the outer leaflet of plasma membranes [28]. Indeed, in normal cells, negatively charged PS are located in the inner leaflet, while the neutral phospholipids, i.e. phosphatidylcholines (PC) and sphingomyelins (SM), are in the outer leaflet of the plasma membrane [29]. However, such condition is reversed in tumors due to the effect of inflammatory cytokines, oxidative stress, acidity, and thrombin that cause loss of membrane asymmetry [30]. Moreover, decreased levels of cholesterol in cancer cells may cause enhanced fluidity of membranes that facilitates AMP-induced apoptosis [31]. The mode of action of host defense peptides is not completely known, however the electrostatic attraction between the negatively charged components of cancer cells and the positively charged AMPs is crucial for strong binding and selective disruption of cancer cell membranes [32,33]. Other mechanisms of action include their ability to selectively interact with ion channels [34].

AMPs represent a new class of anticancer drugs that lack toxicity and may overcome tumor resistance to conventional chemotherapy [35]. The AMP stability in biological fluids and anticancer efficacy is enhanced by different modifications that can be introduced by chemical synthesis, such as the use of D-aminoacid or unnatural amino acids [36,37].

In this review we report the recent advances on AMPs as anticancer agents and we describe their biological activity. In addition, we summarize the methods used to increase their anticancer activity.

## 2. The Classification of Antimicrobial Peptides

AMPs are short molecules evolutionarily well preserved that represent the main innate defense system in plants, invertebrates and vertebrates [38,39,40]. In animals, the AMPs are expressed primarily by the immune system cells such as mast cells, monocytes, and neutrophils [41]. Generally, AMPs contain between 12 and 50 L-aminoacids, have molecular weights ranging from 1 to 10 KDa, are rich in arginine and lysine residues and possess a net positive charge ranging from +2 to +9 at neutral pH [42,43,44,45]. The cationic and amphipathic nature of AMPs allow them to bind negatively charged membranes of bacteria and cancer cells as well as to establish interactions with diverse hydrophilic and hydrophobic components [46].

**Figure 1:**
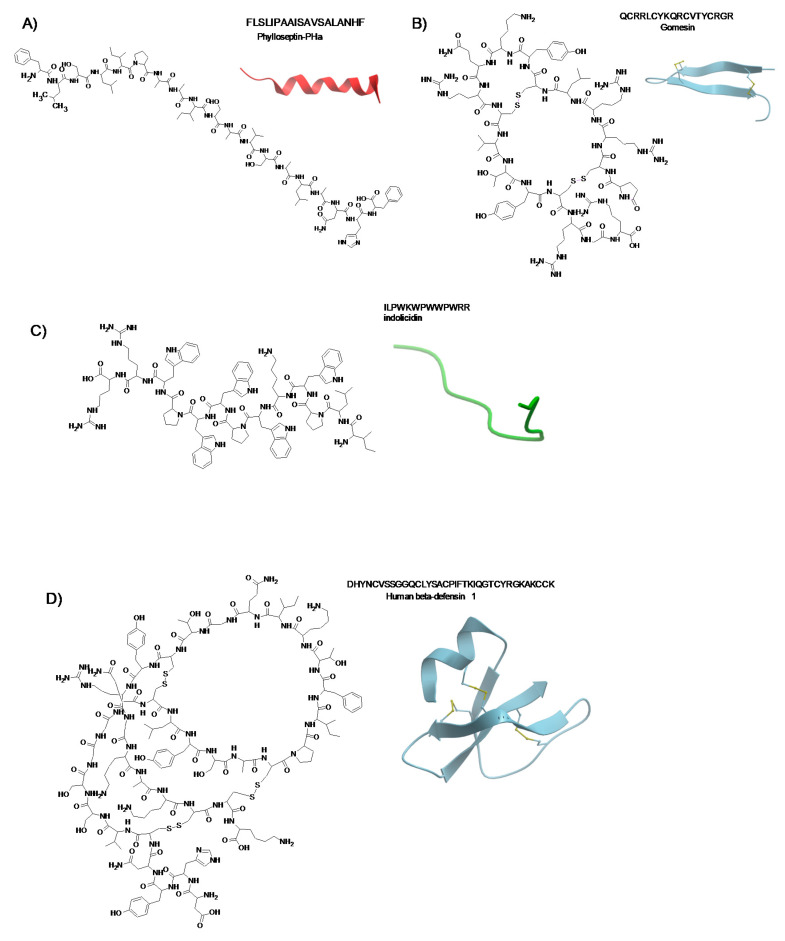
Chemical structures and related secondary structures of the four classes of AMPs: (**A**) Phylloseptin-Pha, α-helix; (**B**) Gomesin, β-strands (two disulfide bounds); (**C**) Indolicidin, unstructured; (**D**) Human beta defensine 1, mixed structure (three disulfide bounds). 3D structures have been generated with ICM-Molbrowser. Physicochemical parameters are reported in http://dramp.cpu-bioinfor.org/ and http://aps.unmc.edu/AP/main.php.

The approximately 2400 AMPs, that have been identified up to now, are classified in four classes according to their structures or in two classes according to their activities [12,47]. Indeed, the AMPs, on the basis of their predicted conformations, are divided in (1) α-helical; (2) β-sheet (β-hairpin and a combination of α-helix and β-sheet); (3) unstructured (neither α-helix or β-sheet); and (4) cyclic peptides (Figure 1). In addition, one group of AMPs comprises those toxic to bacteria and cancer cells, but not lethal to normal mammalian cells, while a second group includes those that are toxic to bacteria, cancer cells as well as normal mammalian cells [36].

The α-helix group comprises the magainins, cecropins, LL-37, temporins, fowlicidins, melittin, while β-sheet peptides include human, plant and insect defensins (type A), plectasin, lactoferricin, tachyplesin, thanatin, and others. The α-helix peptides, containing alanine, leucine and lysine residues to stabilize the helix but not cysteine residues, are the most studied class of AMPs. They have random conformations in aqueous solution and become helical when interact with cell membranes. The β-sheet peptides are rich in proline, glycine, tryptophan, arginine or histidine, and contain from two to ten cysteine residues forming up to five disulphide bridges. Flexible or extended peptides are generally linear, contain infrequent amino acids and include tritrpticin, PR-39, histatins, bactenecins, and indolicidin, the latter being the only molecule that is active towards membranes of pathogenic organisms. Finally, cyclic peptides, such as bacteriocins, acquire the typical conformation through the formation of one or two disulphide bonds [42,48].

### Mode of Action of Antimicrobial Peptides

AMPs have a vast spectrum of activities towards different types of organisms, such as bacteria, viruses, fungi and even mammalian cells, however, the molecular mechanisms by which they act are often not yet clear or well understood [38,49]. The most studied function of AMPs is the killing of microorganisms which may be caused by: (1) destruction of the cell membrane through the extracellular action of peptides depending on specific aminoacid sequences, structure, net charge, amphipathicity, as well as membrane composition [39,47]; and (2) interference of peptides with intracellular pathways following their internalization through an intact membrane [47].

**Figure 2:**
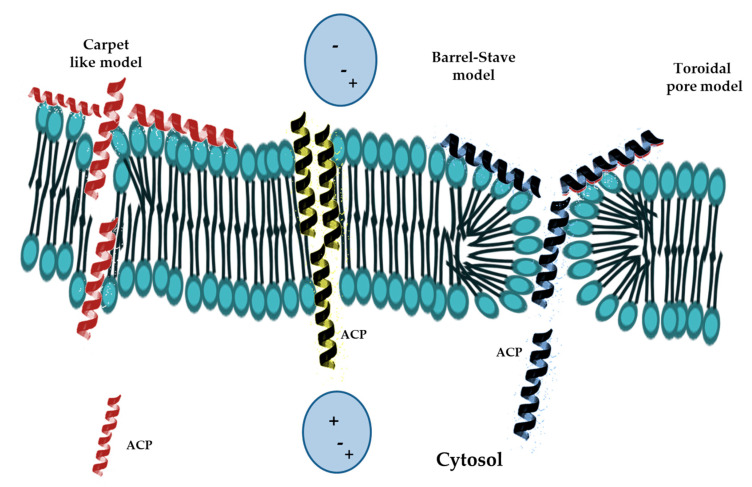
Schematic representation of most significant models of ACP’s action. From left to right: Carpet-like model, Barrel-stave model, Toroidal pore model.

The mode of AMPs interacting with cell membranes has been studied by several techniques, including circular dichroism, X-ray crystallography, nuclear magnetic resonance, reverse-phase high-performance liquid chromatography, and surface plasmon resonance [50,51]. The AMPs have been shown first to destabilize the bacterial envelope and then to interact electrostatically with the anionic part of the membrane. In this stage, the different composition of gram-positive and gram-negative bacteria membranes has shown to influence the cell sensitivity to AMPs. Indeed, the envelope of gram-positive bacteria contains lipoteic, theichuronic, and theicoic acids as well as acidic polysaccharides [42,52]. Instead, the membrane of Gram-negative bacteria contains negative charges originated from the phosphate groups of lipopolysaccharides (LPS) as well as some anionic phospholipids such as cardiolipin and phosphatidylglycerol [53,54]. The positive charged AMPs easily interact with the negatively charged divalent-cation-binding sites of LPS, exposed on the cell surface, and permit the passage of peptides across the outer membrane by a so-called “self-promoted uptake” mechanism [55]. Different models of interaction between AMPs and bacterial membranes have been proposed (Figure 2).

For instance, the “barrel-stave model” consists in the accumulation of monomer peptides on the cell surface followed by conformational changes and aggregation to form barrel-shaped multimers within the bacterial membrane. In this model, the aggregation forces peptides into the hydrophobic center of the membrane and prevents exposure of the hydrophilic parts of the peptide to the hydrophobic parts of the inner membrane. Then, the hydrophobic chains of AMP come into contact with the acylic chains of the membrane, align with the lipid core of the double layer and induce the weakening of the cell membrane. While, the hydrophilic parts of peptides form aqueous pores that widen as the number of aggregated peptides increase [42]. So far, the helical alamethicin is the only AMP known to respond to this model [42,51].

The “carpet-like” model describes the interaction of α-helical positively charged AMPs with the negatively charged phospholipids in the outer layer of the membranes, which are covered by a peptide “carpet”. The AMPs remain parallel to the cell surface without inserting into the lipid bilayers, but when the peptides concentration become critical they rotate on themselves causing phospholipids redirection and increased membrane fluidity. This process weakens the barrier properties of the membrane, destroys the lipid bilayer and causes the formation of micelles [56]. Examples of AMPs fitting this model include cecropin P1, dermaseptin, and LL-37 peptides [42].

The “toroidal pore model” is a two-stage model in which the peptides are inactive at low concentrations and disposed parallel to the bilayer, while they convert to the active form at high concentrations and turn perpendicularly to the bilayer and irreversibly destabilize membranes, while preserving the integrity. The instability of the newly formed toroidal pore allows the entry of peptides into the inner membrane leaflet, whereas the pores disintegration causes the release of peptides into the intracellular space where they inhibit essential pathways, such as DNA replication and protein synthesis, causing bacteria death [56,57]. This model has been studied in some AMPs such as magainin 2, protegrin-1, cecropin A [42].

The “sinking raft model”, similarly to the toroidal pore model, does not show membranolytic activity. In fact, peptides are described to bind the cell membrane and to form a significant curvature which allows the formation of transient pores in the membrane. The “molecular electroporation model” implies that the interaction between peptides, characterized by high charge density and an electrostatic potential of at least 0.2 V, and membranes could generate a difference in the electric potential, allowing electroporation and pore formation.

Hancock et al. proposed the “aggregate channel model” which is based on the binding of peptides to the phospholipidic heads causing the formation of peptide clusters on the membrane [58]. These aggregates, of indefinite and transient nature, associate with water molecules to form channels through which ions and even larger molecules pass directly through, but not cause significant depolarization and destruction of the membranes.

A further model, named “peptide-induced lipid segregation”, has been obtained from the study of the peptide interaction with anionic components of zwitterionic lipids. In this model the binding of AMPs causes the grouping of anionic lipids into separate peptide-lipid domains and the segregation of zwitterionic lipids, producing a rearrangement of the membrane layers with possible significant consequences for cell viability and survival [42,59]. The “leaky-slit” model involves specific peptide–lipid interactions causing fibril formation and toxicity. In this model, AMPs form a linear and amphipathic matrix, upon binding to lipids, with the hydrophobic part facing the double layer peptides. Toxicity is caused by the formation of transient toxic fibrillar oligomers called “leaky slits” which increase membrane permeability due to the highly positive curvature adopted by lipids [59].

The model known as “peptide-mediated non-lamellar phase formation mechanism” is based on the presence of non-lamellar lipid phases in bacterial membranes causing destabilization of the lipid double layer. In this model, AMPs can induce localized changes of the lipid phase by altering the lipid packaging in the double layer. In the inner mitochondrial as well as bacterial membranes the planar bilayers can curve, inducing cubic or other three-dimensionally ordered inverted non-lamellar phases. Some peptides, such as gramicidin S (GS), generate these phases by causing a localized increase in the membrane curvature stress. There is also a possible correlation between non-lamellar phase formation and the activity of cationic amphipathic peptides. Membrane lipids can self-assemble into numerous different phases in aqueous solution, including micellar, lamellar, hexagonal, and cubic phases. Lamellar lipid phases are characterized by two opposing monolayers of lipids with their hydrocarbons chains facing each other. The normal hexagonal (HI) and inverse hexagonal (HII) non-lamellar lipid phases are characterized by lipids forming tubular structures. In this model the lipidic hydrophobic tails form the centre of the HI cylinder, while the core of the HII tube includes the lipidic hydrophilic heads, and the HI and HII hydrophobic tails are outward-facing [38,60].

## 3. Critical Issues, Possible Solutions for Clinical Use of AMPs

Several natural AMPs have critical issues that have hindered their clinical use. Synthetic AMPs may be costly to produce, can be toxic, do not recognize specific receptors hence are not selective, are often unstable in the serum because of proteases, are not bioavailable [61].

The susceptibility to proteolytic degradation can be reduced by changing peptide chirality by replacing natural L-aminoacids with their D-enantiomers which are not recognized by the serum proteases. Cyclic peptides, which have reduced conformational freedom, also decrease the chances of degradation [62,63]. For example, the susceptibility of Polybia-CP, a α-helical peptide with antitumor, antibacterial and antifungal activity, to proteases has been inhibited by replacing lysine with D-lysine (D-Lys-CP). Polybia-CP containing all D-aminoacid residues (D-CP) or only D-Lys-CP both showed a comparable antimicrobial activity but only D-Lys-CP was resistant to the degradation either by trypsin or chymotrypsin [64]. A further strategy that has been used to increase peptide stability is the C-terminal amidation that also protects peptides from protease degradation [54].

Kelly et al. demonstrated that AMP pegylation was also able to inhibit the proteolysis. Indeed, a pro-peptide obtained by covalent binding of AMP P18 to a linker containing the catalytic sites of cathepsin B was thermodynamically stabilized by the addition of polyethylene glycol (PEG) [65]. Another possibility could be the use of a liposomal formulation to wrap a inactive pro-peptide AMP in a protecting liposome [66,67]. In 2018, Wu et al. demonstrated that liposomal formulation increased the administration efficiency of the HCC-targeting peptide SP94 (SFSIIHTPILPL) in a HCC xenograft mouse model. Particularly, SP94-mediated targeting improved anti-tumor efficacy by enhancing pharmacokinetics and tissue distribution, allowing the accumulation of a vast amount of anti-cancer drug in the tumor [68]. On the other hands, liposomes have the disadvantage of being not specific to cancer cells and to vehiculate AMPs into mitochondrial membranes of normal cells causing damaging. Only if liposomes are tagged with ligands specific to tumor cells they may become safe for human in vivo testing [69].

Domalaon et al. modified two cationic tripeptide sequences (KKK and KGK) by introducing fatty acid chains, containing between fourteen and twenty carbon chains to confer amphiphilicity, and evaluated their activity against breast, prostate and pancreas epithelial cancer cell lines. The lipotripeptide C16-KKK-NH_2_ and lipotetrapeptide C16-PCatPHexPHexPCat-NH_2_ ((L-4*R*-aminoproline (PCat), L-4*R*-hexyloxyproline (PHex)) showed diverse anticancer activities such as caspase-mediated apoptosis and caspases independent cell death, respectively. The difference between the two moieties, besides the cationic and amphiphilic properties, was in the secondary conformation with the polyproline peptide having a more rigid structure [70].

The use of bacteriophages as peptides carriers has also been experimented by Dąbrowska et al. that observed a decreased tumor growth by injecting phages expressing the anticancer TyrIleGlySerArg (YIGSR) peptide in to the peritoneum of BALB/c mouse engrafted with 4T1 tumor cells [71]. Hao et al. have conjugated the cell-penetrating peptide TAT to the C-terminus of HPRP-A1 peptide and obtained enhanced specificity against cancer cells [72]. The use of nanoparticles is a further example of delivery strategy optimization [73]. Pazos et al. in 2016, designed a novel amphiphilic peptide with aldehydic properties which was capable to reduce silver ions and to nucleate silver nanoparticles in water. The spontaneously generated monodisperse silver particles along with the filamentous organic structures showed antimicrobial activity and low toxicity toward eukaryotic cells [74]. Melittin, isolated from bee venom, is a peptide with antimicrobial and anti-cancer properties that causes hemolysis of cancer cells, but when administered intravenously can cause severe allergic side effects. 

The encapsulation of melittin in Poloxamer 188 produces nanoliposome formulations that showed decreased inflammatory allergic reactions and toxicity in mice, while preserving anti-tumor activity such as growth suppression of subcutaneous and orthotopic hepatocarcinoma implants in mice. The anti-tumor activity of the new formulation is comparable to the FDA-approved sorafenib in hepatocarcinoma studies [75,76].

Metal-based nanoparticles (MNPs) are colloidal particles that show specific characteristics such as optical behavior, electrical conductivity, and high thermal and chemical stability. MNPs, particularly silver (Ag) and gold (Au) nanoparticles (NPs), have a strong antimicrobial potential and can be internalized specifically by cancer cells, avoiding toxicity to healthy cells [77]. Intravenous MNPs administration exposes the moieties to mononuclear phagocytes causing the activation of the innate immune system through suppression of the Toll-like receptor and induction of interleukin-1β (IL-1β) and IL-18 secretion. Thus, MNPs stimulate the cellular and humoral immune response to produce pro-inflammatory cytokines (IL-1, IL-6, and tumor necrosis factor α (TNF-α)) and immunosuppressor cytokines (IL-10 and transforming growth factor β (TGF-β)), preventing the excessive and harmful proinflammatory responses including sepsis. Cationic AMP/MNPs can selectively bind and specifically recognize transformed cancer cells. Moreover, Ag-MNP and Au-MNP peptides are more stable, more effective against cancer cells and less toxic to normal cells [78,79]. Indeed, the cathelicidin LL-37 and its peptidomimetic analog ceragenin CSA-13 showed an increased anticancer activity against colorectal cancer cells if adsorbed on the MNP surfaces. Moreover, the MNP-CSA-13 showed a pro-apoptotic activity stronger than MNP-LL-37 on colon cancer cells [80]. More recently, Piktel et al. observed that MNP-CSA-13 reduced the viability and inhibited proliferation of MCF-7 and MDA-MB-231 breast cancer cells through the disruption of the oxidative balance. The ultimate result of the increased reactive oxygen species (ROS) generation is cell membrane disorganization and induction of caspase-dependent apoptosis via mitochondrial membrane depolarization [81].

Nanoformulations as delivery systems for AMPs have only been evaluated in experimental animal models. However, intense development is now ongoing, including scaling up and quality assurance of nanocarriers to bring these products into clinical phases, but so far there are no FDA-approved clinical trials [75,82].

Finally, among the other issues, the cost of production could be reduced by large-scale engineered manufactures. Cancer cell recognition not mediated by receptors, but by lipids, could even be considered an advantage not a weakness. AMPs are less likely to induce resistance to pathogens or transformed cells than current antibiotics or cancer drugs [54].

### 3.1. Antimicrobial Peptides as Anticancer Drugs

Many AMPs, acting as anti-cancer peptides (ACPs), have the ability to cross cell membranes and to kill either bacteria or cancer cells (Table 1). The specific recognition of tumor cells is facilitated by the presence of the negatively charged PS on their surface, caused by high levels of ROS and hypoxia that modify tumor microenvironment and induce membrane phospholipids dysregulation [47]. Indeed, tumor cells lose the asymmetry of phospholipids distribution between the outer and inner layers of the plasma membrane and expose PS on the outer layer of tumor cells [83].

Several factors contribute to the increase of negative net charge on cancer cell membranes, compared to normal cells, such as the over-expression (9%) of PS, increased levels of zwitterionic phosphatidylethanolamine molecules, deregulated glycolipids glycosylation and membrane glycoproteins with repeated regions of O-glycosylation as well as over-expression of heparan sulfate proteoglycans. These phenomena occur both in the vascular endothelium and in the epithelial tumor tissues and are associated with tumor progression and survival. Accordingly, prostate, breast, lung, pancreas as well as skin carcinomas and colorectal cancers show an over-expression of membrane mucins and proteoglycans [47,117]. Such conditions increase the electrostatic interaction of ACPs with the surface of cancer cells. Other features, such as lower cholesterol content, the presence of filopodia and microvilli on the tumor cells surface may enhance their susceptibility to ACPs and can promote selective cytotoxic activity [42]. An increase in the number of microvilli on the tumor cell surface enhances the extension of contact area compared to healthy cells and increases the attraction of ACPs to cancer cells [118]. The fluidity and stiffness of tumor cell membranes are compromised by the internalization of ACPs into the hydrophobic layer facilitating their cell lytic effect [38].

### 3.2. Examples of ACPs

#### 3.2.1. Cathelicidins

Cathelicidins, encoded by the CAMP gene, are α-helix peptides representing the main class of ACP in mammalians. They are highly conserved N-terminal peptides, identified in macrophages and polymorphonuclear leukocytes (PMNs), that contain a catheline domain and an antimicrobial cationic peptide derived from the extracellular proteolysis of the C-terminal sequence. Cathelicidins exert a membranolytic activity towards tumor cells. The leucine-leucine-37 (LL-37; named for the first two amino acids and with a total of 37 amino acids), derived from the cleavage of the human cationic antimicrobial peptide 18 (hCAP-18; 18 for its approximate molecular weight of 18 kDa) by proteinase 3, plays a crucial role in the adaptive immunity, growth inhibition, chemotaxis, and wound healing [119,120,121]. The LL-37 destabilizes specifically cancer cell membranes by a “toroidal pore” mechanism [122]. Indeed, it has been shown to induce permeabilization of U937 cell membranes with a low hemolytic activity towards erythrocytes [47,87].

LL-37 generates caspase-independent calpain-mediated apoptosis in Jurkat cells as well as mitochondrial depolarization and caspase-independent apoptosis in human oral squamous cell carcinoma (OSCC) cells SAS-H1 [86]. At the same time, LL-37 is unable to cause cell death of keratinocyte cell line HaCaT, showing that it can be specific and selective for the treatment of OSCC [85].

Immunohistochemical staining of tissue arrays revealed that LL-37 is highly expressed in normal colon mucosa, while it is downregulated in colon cancer tissues because of DNA methylation in the CAMP gene promoter. The addition of LL-37 in cancer cells induces caspase-independent apoptosis, DNA fragmentation and PS externalization as well as nuclear translocation of the apoptosis-inducing factor (AIF) causing overexpression of BAX and BAK and p53-mediated downregulation of Bcl-2 [123]. Moreover, the LL-37 peptide has been described to have an indirect antineoplastic activity, through its ability to modify stromal cells and tumor microenvironment, without directly affecting the viability of tumor cells. Cheng et al. demonstrated that cathelicidin inhibits proliferation of fibroblast-supported colon cancer cells either by suppressing the tubulin distribution in colon fibroblasts or by hindering the epithelial-mesenchymal transition (EMT) of colon cancer cells [88].

Since 2016, LL-37 is under testing in a phase I–II clinical trial to evaluate its efficacy against melanoma following intratumor injection in patients with no immunodeficiency syndromes and with surgically unresectable lesions of at least 1 cm in diameter (Table 2), https://clinicaltrials.gov/ct2/show/NCT02225366 [54].

#### 3.2.2. Human Defensins

The human neutrophil peptides (HNP-1, HNP-2 and HNP-3), also named α-defensins, have been shown to form dimers on cell membranes and to possess cytolytic activity against several cell lines including the U937, a pro-monocytic human myeloid leukaemia cell line, the K562, a human erythroleukemic cell line, and IM-9 and WIL-2 lymphoblastoid B cells [124]. Furthermore, increased levels of HNP-1 have been shown to induce apoptosis in tumor cells either by an extrinsic (cytoplasmic) pathway, involving the Fas death receptor that is member of the TNF receptor superfamily, or by an intrinsic (mitochondrial) pathway, via the release of cytochrome c from the mitochondria and activation of death signals [89,90]. HNP-1 has also shown to be involved in the immune response against human papillomavirus (HPV)-associated cervical neoplasia through the inhibition of angiogenesis [125]. Human β-defensin-3 (hBD3) has a net charge of +11 and displays anti-tumor activity against HeLa, Jurkat and U937 cancer cell lines, mediated by its binding to the phosphatidylinositol 4,5-bisphosphate [PI(4,5)P2] on cell membrane and cytolysis [91].

#### 3.2.3. Bovine Lactoferricin (LfcinB)

Bovine lactoferricin (LfcinB) is a well-known ACP, derived from the pepsin-mediated hydrolysis of the natural milk iron-binding glycoprotein lactoferrin [126]. The LfcinB is a 25-mer peptide that adopts two amphipathic structures, a loop peptide generated by two Cys disulphide bond and a twisted antiparallel β-sheet structure, both toxic to cancer cells, but with the cyclic version more active than the linear analogue [92,93]. Being demonstrated that the activity depended on the amphipathic secondary structure, the nature, size, and positioning of aromatic amino acids, new cationic 9-mer oncolytic peptides were designed based on the more active structure of LfcinB. One of them, LTX-315, showed a significant antitumor activity towards a large panel of both drug-resistant and drug-sensitive cancer cells and reduced activity against normal cells [114]. LTX-315 was shown to induce cell death by a dual effect, namely cytolysis and immunogenicity. In fact, Frank et al. demonstrated that tumor cell lysis released danger-associated molecular patterns (DAMPs) and high mobility group box protein 1 (HMGB1) which were able to trigger immune response against cancer cells [127]. HMGB1 has been recognized to activate the immature dendritic cells and to generate tumor-specific cytotoxic T-lymphocytes against A20 lymphoma cells causing tumor cell lysis. Treatment with LTX-315 produced remission in mice and protected animals from the second syngeneic tumor, but not from a different type of tumor, demonstrating the specificity of the immunogenic cell death. Thereafter, LTX-315 has been evaluated in a phase I in human clinical study and demonstrated to cause changes in the tumor microenvironment, increase of T effector cells, decrease in immunosuppressive cells, and finally tumor necrosis. For these characteristics, the LTX-315 is considered an elective drug to be combined with immune checkpoint inhibitors (anti-CTLA4/anti-PD-1) [115]. A phase I “open-label, multi-arm, multi-centre, multi-dose, dose-escalation study” (NCT01986426), exploring the LTX-315 efficacy as monotherapy or in combination with either ipilimumab or pembrolizumab in patients with transdermally accessible tumors, started on 2013 and is still open (last updated December 2016) [54]. A Phase II clinical trial evaluating the intratumor administration of the LTX-315, followed by treatment with tumor infiltrating lymphocytes (TILs), in patient with advanced metastatic soft tissue sarcoma is also ongoing (Table 2).

#### 3.2.4. Gomesin

Gomesin (Gm), derived from a Brazilian spider, *(Acanthoscurria gomesiana)* has demonstrated to have anti-cancer activity in vitro against a broad range of murine and human cancer cell lines, including melanoma and leukemia derived cell lines [128]. The mechanism involves the interaction of Gm peptide with membrane lipids and the formation of unilamellar giant vesicles (GUV), containing mixtures of neutral lipid-neutral palmitoyl oleoyl phosphatidylcholine (POPC) and negative lipid-loaded palmitoyl oleoyl phosphatidylglycerol (POPG) or cholesterol, thus imitating the behaviour of bacterial and mammalian cell membranes, respectively [94]. Gm disrupts the cell membranes via the carpet model, but the factors responsible for cell-specific anti-cancer activities of Gm and other ACPs remain unknown. Some factors, such as gangliosides, heparin sulphate, cholesterol, and PS levels, may have a role in such specificity but has not yet been demonstrated. Gm has shown to have a low activity against cervical cancer derived cell lines [129].

#### 3.2.5. Mastoparan-C

Mastoparan-C (MP-C), extracted from the venom of the European hornet (*Vespa crabro*), and its analogues, that have been optimized for stability and membrane permeability by cys-cys cyclization and a N-terminal TAT extension, showed anti-cancer activity against non-small cell lung cancer H157, melanocyte MDA-MB-435S, human prostate carcinoma PC-3, human glioblastoma astrocytoma U251MG and human breast cancer MCF-7 cell lines. Importantly, all three peptides showed a relatively weak activity against the normal human microvascular endothelial cell line HMEC-1.

The link of TAT peptide to the MP-C N-terminal domain dramatically enhances the anti-cancer properties. Indeed, TAT is a short cell-penetrating peptide that facilitates the intracellular peptide delivery, the interaction of MP-C with the mitochondrial membrane phospholipids thus inducing apoptosis, selective activation of phospholipase, inhibition of ATPase activity, and enhancement of tMP-C anti-proliferative properties in B16F10-Nex2 melanoma cells [130]. However, the chemical modifications have not improved stability and cancer cell specificity hence the tMP-C shows severe cytolytic effects on normal human cell line HMEC-1. More studies are needed for future clinical application [95].

#### 3.2.6. Cecropin B1

Cecropin B1 (CB1), derived from cecropin of the silkworm *Hyalophora cecropia* has significant anti-proliferative effect against cancer cells with low cytotoxic activity against normal cells. The CB1 showed higher tumor growth inhibition than docetaxel in the NSCLC cell line NCI-H460 xenografted in mice. The mechanism is based on the interaction between the peptide amphipathic residues and the cell membrane hydrophilic and hydrophobic moieties causing pore formation and apoptosis [96,97].

#### 3.2.7. Magainin 2

Magainin 2 (MG2) was isolated from the African clawed frog (*Xenopus laevis*) and recognized to have anti-tumor activity in human lung cancer cells A59 and in Ehlrich’s murine ascites cells [98]. Recent studies have also shown that MG2 may represent a novel anti-cancer strategy being cytotoxic and anti-proliferative in bladder cancer cells, through the formation of pores on cell membranes, but has no effect on normal human or murine fibroblasts [99]. Some synthetic derivatives, including MSI-136 and Pexigananan MSI-78 as well as analogs containing D-aminoacids, exhibited higher cytotoxic activity than MG2 due to increased amphipaticity of the α-helix structure compared to MG2 [131].

Liu et al. linked the MG2 to Bombesin (MG2B) and observed that the conjugated compound has higher cytolytic effect on tumor cells compared to MG2, especially in vivo in mice engrafted with MCF-7 breast cancer cells [132]. In addition, Shin et al. demonstrated that the hybrid obtained by the link of Cecropin A and MG2 (CA-MA-2, KWKLFKKI-P-KFLHSAKKF-NH_2_) has a significant high anti-cancer activity against several tumor cell lines and low toxicity to erythrocytes and NIH-3T3 primary fibroblasts. Furthermore, the 50% growth inhibition (IC50) against several tumor cell lines was obtained with a lower concentration than that used for fibroblasts. CA-MA-2 induced disruption of large PC/PS-based unilamellar (mixed PC-PS) vesicles indicating a membrane perturbation mechanism [100].

#### 3.2.8. Buforin IIb

Buforin IIb is a histone H2A-derived antimicrobial peptide, a synthetic analog of buforin II derived from buforin I, that was isolated from the stomach tissue of the Asian toad *Bufo bufo garagrizans*, Buforin IIb has a α-helical sequence at C terminus and interacts with gangliosides of cancer cells surface causing destruction of membrane and mitochondria-dependent apoptosis. Buforin IIb induces cytotoxicity against a large panel of cancer cell lines such those derived from leukemia, breast, prostate, and colon cancer [101].

#### 3.2.9. Brevinin-2R

Brevenin-2R is a defensin isolated from the skin of the frog *Rana ridibunda* which has a non-hemolytic cytotoxicity against several transformed cells, such as T-cell leukaemia Jurkat, B-cell lymphoma BJAB, colon carcinoma HT29/219 and SW742, fibrosarcoma L929, breast adenocarcinoma MCF-7 and lung carcinoma A549 cells compared to primary cells including peripheral blood mononuclear cells, T cells, and human lung fibroblasts. The hypothetical toxic mechanism is based on a lysosomal death pathway (LDP) demonstrated by the fact that inhibitors of lysosomal membrane permeabilization prevented brevinin 2R from inducing cell death. Ghavami et al. demonstrated that the treatment with brevenin 2R induces LDP and autophagy-like cell death through the formation of autophagosomes. Moreover, during the early stage of cell death brevenin 2R causes decreased mitochondrial membrane potential and increased ROS levels, without caspase activation or release of apoptosis-inducing factor or endonuclease G [102].

#### 3.2.10. *Limnonectes fujianensis* Brevinvin

*Limnonectes fujianensis* brevinvin (LFB), a novel AMP identified in the skin of the frog *Limnonectes fujianensis*, is an amphipathic, hydrophobic, α-helical, and β-turn peptide that is able to penetrate the lipidic bilayer causing cell death. Li et al. demonstrated that LFB was specifically active on the large cell lung cancer H460, the melanoma cell line previously described as a breast cancer MDA-MB-435S, the glioblastoma U251MG, and the colon cancer HCT116 cell lines, with the latter type of cells being more sensitive to the treatment. In fact, LFB reduced the proliferation rate of HCT116 cells in a dose-dependent manner and at high concentrations caused cell death through membrane disruption, without inducing apoptosis. Lastly, LFB exhibited significant hemolytic activity [103].

#### 3.2.11. Phylloseptin-PHa

Phylloseptin-PHa (PSPHa) is a newly discovered peptide extracted from the skin of the frog *Pithecopus hypochondrialis* of the *Phyllomedusinae* family. PSPHa and its analogs PHa1-5 reduced the viability of breast cancer cells MCF-7 as well as of non transformed breast epithelial cells MCF10A, however the PSPHa was less active than its analogs with a little more effect on MCF-7 than the MCF10A cells [104].

#### 3.2.12. Ranatuerin-2PLx

Ranatuerin-2PLx (R2PLx) is a 28-amino acid polypeptide derived from the skin of the pickerel frog (*Rana palustris)*. The anti-cancer activity of R2PLx was demonstrated by the treatment of prostate cancer cell PC-3 which induced AMP-dependent caspase-3 and early cell apoptosis. R2PLx was demonstrated to be more potent against cancer cells than against bacteria and selective against cancer cells. The mode of action of this peptide does not seem to be related to membrane-lytic activity [105].

#### 3.2.13. Dermaseptins

Dermaseptins (DRS) are polycationic peptides isolated from *Phyllomedusa* frogs that are effective against bacteria, parasites, protozoa, viruses, and cancer cells in vitro. The antimicrobial and anti-cancer activity involves the destruction of cell membranes through a carpet-like model based on their binding to the outer phospholipid layer which causes the pores formation and internalization between the phospholipid heads [106]. Dos Santos et al. reported that dermaseptin B2 (DRS-B2) appears to be active towards the sulphated glycosaminoglycans (GAGs) present on the surface of PC3 prostate cancer cells membranes [107]. Zhu et al. demonstrated that adding the TAT peptide to the N-terminal domain increased the interaction between the ACP and GAGs present on tumor cell membranes [133]. Such interactions may cause greater fluidity of tumor cell surfaces and increased destabilization thus influencing the receptors link and communications between the environment and the tumor cells [36]. DRS-B1-B2, DRS-S1-S5 DRS-O1, DRS-CA1 DRS-DU1 are effective against fungi in vitro demonstrating cytotoxicity on *Candida albicans* cultures, with DRS-S3 triggering apopotosis [106]. The DRS-S4 and DRS-9 possess antiviral activities against HPVs, associated with genital infection and cancer, and HSV by interfering with viral and cellular factors in the early phase of viral replication. Moreover, DRS-S4 and DRS-S9 were shown to inhibit HIV-1 attachment to endometrial cells, the uptake by dendritic cells and subsequent viral spread to T-cells. DRS-S4 has shown to be effective against Rabies virus in mice [106,134].

The novel dermaseptin-PT9 (DPT9) peptide was isolated from the skin of the frog *Phyllomedusa tarsius*. The chemically synthesized DPT9 has been demonstrated to be effective against a broad group of the microorganisms through the disruption of cell membranes and to have a weak hemolytic activity towards horse erythrocytes. In addition, its analog K8, 23-DPT9, in which Asp8 and Glu23 were substituted by lysine residues, displayed high anti-proliferative activity against cancer cells, and weak activity against the normal human microvascular endothelial cell line HMEC-1. Both DPT9 and K8, 23-DPT9 peptides showed an increased ability to damage the cell membrane of human lung cancer cell lines NCI-H125 and H157, human prostate cancer cell line PC-3 as well as the human pancreatic cancer cell line PANC-1. The anti-tumor action of the peptides is related to a necrotic-like pathway involving cell membrane damage, alteration in the mitochondrial membrane potential and activation of caspase-3 [108].

#### 3.2.14. Chrysophsins

Chrysophsin-1,-2 and-3 are cationic amphipathic, α-helical AMPs that were isolated from the gill cells of red sea bream (*Chrysophrys major*). The AMP chrysophsin-1 is able to disrupt the plasma membrane of several cancer cell lines at much lower concentrations compared to the CA-MA-2 peptide [109]. In vitro studies showed that chrysophsin-1 had a significant inhibitory effect against human fibrosarcoma HT-1080, histiocytic lymphoma U937, and cervical carcinoma HeLa cell lines. Chrysophsin-1 does not affect caspase expression therefore it does not induce cell death via apoptosis.

#### 3.2.15. Ss-arasin

The Ss-arasin is a 65 amino acid long pro-AMP isolated from the mud crab, *Scylla serrata*. The 41 amino acids mature peptide contains a N-terminal Gly/Arg-rich domain which may function as a signal sequence directing the newly synthesized protein toward the secretory pathway. A recombinant rSs-arasin was shown to inhibit human cervical carcinoma HeLa and colon carcinoma HT-29 cell growth. Further molecular studies are required to characterize the mechanisms by which rSs-arasin exerts cytotoxicity against cancer cells [110].

#### 3.2.16. Turgencin A and Turgencin B

Turgencin A and turgencin B are two novel antibacterial peptides recently isolated from the Arctic marine colonial ascidian *Synoicum turgens*. The peptides contain an amidated C-terminus, unusual disulfide bridges connecting six cysteines methionine residues conferring different degrees of oxidation. The turgencin AMox1 containing one oxidized methionine is the most potent AMP and displayed antimicrobial activity against Gram-negative and Gram-positive bacteria as well as inhibited the growth of melanoma cancer cells A2058 and the human fibroblast cell line MRC-5 [111].

#### 3.2.17. D-K6L9

D-K6L9 is an engineered membranolytic anticancer peptide made of only lysine and leucine aminoacids. Papo et al. demonstrated selective binding of D-K6L9 to negatively charged PS and induction of cell membrane depolarization. The K6L9 D-enantiomer is much more stable than the K6L9 peptide containing L-aminoacids [112]. The D-K6L9 administration to immunodeficient mice, implanted with breast and prostate cancer cell lines showed reduced neovascularization. Moreover, the direct administration of D-K6L9 and glycyrrhizin, a HMGB1 protein inhibitor, into B16-F10 murine melanoma tumors inhibited the growth but only during the period of the treatment and did not enhance animal survival [135].

#### 3.2.18. KLA

The (KLAKLAK)2 amphipathic α-helical peptide is an ACP causing distortion of the mitochondria membranes thus promoting apoptosis. Jakel et al. showed that (KLAKLAK)2 was effective at killing human breast cancer and other tumor cells while it was non-toxic to healthy mammalian cells [136]. Horton and Kelley demonstrated that D-(KLAKLAK)2 exhibited a mitochondrial localization in HeLa cells causing mitochondrial membrane disruption, which led to mitochondrial contents leakage into the cytoplasm as well as release of cytochrome c and consequent activation of apoptosis-inducing factor-1 and downstream caspases inducing apoptosis [137]. Furthermore, the treatment of nude mice bearing MDA-MB435S breast cancer tumors with (KLAKLAK)2 for 60 days showed that those receiving peptide survived 16 days longer in comparison to the control mice [113]. Bahmani et al. studied the synergistic effect of gamma-irradiation and (KLAKLAK)2 administration delivered by a cell-penetrating peptide (CPP) into radioresistant human monocytic leukemia cells THP-1. Radioresistant THP-1 cells cultured with KLA-CPP and then exposed to ionizing irradiation exhibited reduced viability and increased cell apoptosis [138].

Bouchet et al. evaluated the anti-cancer effect of D-(KLAKLAK)2 bound to the Asn-Gly-Arg (NGR) motif, which is known to recognize the CD13-positive blood vessels in tumors but not to CD13 expressed in normal epithelial tissues. This selectivity might be related to different glycosylation or conformations of the CD13 isoforms. Therefore, targeting cells with NGR- drugs doxorubicin, 5′ fluoro-2′-deoxyuridine, human cytokines (TNF-α and IFN-γ), and the NGR-D-(KLAKLAK)2 peptide might facilitate drug delivery to various solid tumors and tumor-associated angiogenic blood vessels. The authors showed that CNGRC-GG- D-(KLAKLAK)2 peptide induced death of leukemia cell lines U937, THP-1, NB4 and HL-60 as well as of primary blood cells from leukemic patients through a caspase-independent mechanism, without DNA fragmentation, featuring PS externalization and membrane disruption [139]. TAT-RasGAP317−326 is a peptide composed of the TAT HIV 48–57 sequence, and ten amino-acid sequence derived from the Src Homology 3 Domain (SH3 domain) of p120 RasGAP. This peptide can induce cancer cell death directly without inducing apoptosis. TAT-RasGAP317−326 inhibits metastatic progression [116].

#### 3.2.19. Dusquetide

Dusquetide (SGX942) is a pentapeptide (RIVPA) belonging to the Innate Defense Regulator (IDR) class. SGX942 modulates the innate immune response to both pathogen-associated molecular patterns (PAMPs) and damage-associated molecular patterns (DAMPs) through its binding to p62, a key adaptor protein that triggers innate immune activation by acting downstream the key sensing receptors, such as toll-like receptors [140,141]. Dusquetide has been shown to be effective in the treatment of severe oral mucositis (SOM) affecting head and neck cancer patients undergoing chemoradiation therapy. Dusquetide is under evaluation in a Phase III clinical trial (NCT03237325) (Table 2).

### 3.3. ACP for the Treatment of Hepatocellular Carcinoma

Hepatocellular carcinoma (HCC) is a major cause of cancer-related death. Main risk factors are represented by alcohol abuse, chronic infection with hepatitis B and C viruses, metabolic syndromes such as non-alcohol fatty liver disease (NAFLD) or aflatoxin intake. Recently, several ACPs directed against HCC have been studied and the activity of some of them discussed herein (Table 3).

#### 3.3.1. Tv1

Tv1 (TRICCGCYWNGSKDVCSQSCC) is a venom peptide derived from the predatory marine snail *Terebra variegate* that has been demonstrated to be selectively cytotoxic to murine liver cancer cells. The mechanism is based on the ability of Tv1 to cause down-regulation of the cycloxygenase-2 (COX-2) and modulation of transient receptor potential channels (TRPC), mainly TRPC6 and TRPV6. The expression and activity of TRPCs has been suggested as marker and promoter of cancer progression [142]. Recent reports suggested that altered expression or function of TRPC6 and other TRPCs in liver tumors and derived cell lines might play a role in the development, progression, and metastasis of HCC [143]. Anand et al. evaluated the effect of Tv1 in vitro in the mouse liver cancer cells 1MEA and in vivo in the Balb/c mice implanted with 1MEA and observed that the peptide induced apoptosis by inhibiting the calcium influx into tumor cells via TRPCs [144].

#### 3.3.2. SALL4 Peptide FFW

SALL4 peptide FFW (RRKFAKFQWI) is derived from the Sal-like 4 nuclear factor that plays central roles both in the maintenance of stem cell pluripotency and in HCC progression. The FFW antagonizes the interaction between SALL4 and the nucleosome remodeling deacetylase (NuRD). The SALL4 complexed to RBBp4, the chaperone subunit of NuRD, causes silencing of the tumor-suppressor gene PTEN in hepatocellular carcinoma. Liu et al. demonstrated that FFW has a strong anti-tumor activity through the inhibition of the repressive function of SALL4, which is highly expressed in leukemic malignancies and most solid tumors, including HCC [145].

#### 3.3.3. SP94

The HCC targeting peptide, SP94 (SFSIIHTPILPL) has been identified by phage-display technology and shown to have a high and selective affinity for s human HCC cell lines, such as Mahlavu and SK-HEP-1, with a minimal effect against normal hepatocytes and other tissues [146]. To date, the antigen recognized by SP94 has not yet been identified, however immune histochemical techniques demonstrated that SP4 is expressed in HCC biopsies as well as in normal liver tissue. Recently, new formulations have been developed by combining the SP94 with drug delivery systems, including mesoporous silica nanoparticle-supported lipid bilayers, virus like particles (VLPs) such as the bacteriophage MS2 as well as the ^99m^Tc/^188^ReHYNIC-SP94, that is a probe targeting SP4 to HCC cells for imaging and therapy [153,154,155]. Moreover, the potential of SP94 as drug delivery system by its conjugation with doxorubicin-encapsulated liposomes has also been assessed [146].

Wu et al. confirmed the tumor-targeting properties of SP94 peptide by comparing the efficacy of non-targeted PEGylated liposomal doxorubicin (LD) versus SP94-conjugated PEGylated liposomal doxorubicin (SP94-LD). They demonstrated that in the HCC xenograft mouse model the SP94-LD accumulated significantly in HCC tumors with low toxicity to normal cells and that the SP94-LD plasma pharmacokinetics was similar to LD [68].

#### 3.3.4. R-Tf-D-LP4

R-Tf-D-LP4 (KWTWKNSNGATWALNVATELKKEWTWSHRPYIAH) is a cell-penetrating peptide derived from the voltage-dependent anion channel 1 (VDAC1), which is a beta-barrel protein located in the outer mitochondrial membrane. This protein mediates the metabolic cross-talk between the mitochondria and other cell compartments. VDAC1 becomes active, following the interaction with other factors, and plays a crucial role in the control of cell survival and death being able to regulate several cellular processes, including ATP rationing, Ca^2+^ homeostasis and apoptosis execution [156,157]. The R-Tf-D-LP4 sequence contains D-amino acids and a recognition sequence (Tf, HAIYPRH) derived from the human transferrin receptor (hTfR).

Pittala et al. demonstrated that R-Tf-D-LP4 dramatically inhibits tumor growth in three different HCC mouse models. The authors observed that R-Tf-D-LP4 induced cell energy perturbation and antagonized VDAC1 for the interaction with Bcl- 2, Bcl-xL and hexokinase II (HK) thus inducing apoptosis in diethylnitrosamine-induced HCC, metabolically (high-fat diet-32) induced HCC, and subcutaneous Hep-G2 cell xenograft mice models. The R-Tf-D-LP4 peptide, besides apoptosis, caused the attenuation of processes associated with liver cancer microenvironment, as well as steatosis, inflammation, and fibrosis. Other effects included the diminished activation of liver stellate cells and their differentiation/activation into myofibroblasts-like cells [147].

#### 3.3.5. GG-8-6

The GG-8-6, cyclo-VLPILLVL compound 1, was synthesized based on the lead compound grifficyclocin B, a cyclic peptide extracted from a *Goniothalamus* species (*Annonaceae*) plant. Grifficyclocin B as well as the GG-8-6 and its twelve analogues showed anti-tumor activity against human HCC cell lines SMMC-7721 and HepG2 based on the induction of apoptotis, activation of caspase and a G2/M cell blockage. These peptides were able also to inhibit tumor growth in vivo in a mouse xenograft tumor model [148].

#### 3.3.6. β3 Peptide

The cyclic β3-peptide trimers DLYYLMDLSYSMKGGDLYYLMDLSYSMKGGDLYYLM-DLSYSMK represent a new class of self-assembling transmembrane ion channels that make flat, ring shaped structures and form tubular channel structures through a backbone-backbone hydrogen bonding. Wang et al. observed that the β3 peptides were able to inhibit tumor cell adhesion to the extracellular matrix and to prevent tumor metastasis in the highly metastatic HCC cell line HCCLM6 and fibronectin model. The treatment with β3 peptide of nude mice transplanted with LCI-D20 HCC tumors caused inhibited of tumor recurrence and prolonged survival time [149].

#### 3.3.7. CecropinXJ

CecropinXJ (RWKIFKKIEKMGRNIRDGIVKAGPAIEVLGSAKAIGK) is a cationic AMP isolated from the larvae of domestic silk moth, *Bombyx mori*. Xia et al. observed that CecropinXJ inhibits the growth of hepatocellular carcinoma Huh-7 cells in a dose- and time-dependent manner, by inducing cell cycle arrest in S phase and apoptosis by caspase-3. The authors showed that this peptide downregulates the expression of Bcl-2 gene and upregulates the Bcl-2-associated death promoter as well as the Bcl-2-associated X protein [150].

#### 3.3.8. GW-H1

The GW-H1 (GYNYAKKLANLAKKPANALW) peptide is a novel cationic amphipathic AMP effective against several HCC derived cell lines, including J5, Huh7, and Hep3B. Chen et al. showed by flow cytometry and western blot analysis that GW-H1 anti-tumor activity is based on its ability to induce apoptosis in a dose-dependent manner. The down-regulation of Hsp27, phosphoglycerate kinase 1, and triosephosphateisomerase in GW-H1 treated cells confirmed the results. Moreover, GW-H1 was shown to inhibit the metastatic progression of J5 HCC cells. The activity of GW-H1 peptide relies on its attachment to cell membranes and migration into the nucleus or other organelles [151].

#### 3.3.9. Bombinin

Bombinin peptides have been recently identified in a skin secretion-derived cDNA library of the Oriental fire-bellied toad, *Bombina orientalis.* A single transcript was shown to encoded full-length peptide precursor producing two novel peptides: (1) the bombinin-like peptide (BO1) (GIGSAILSAGKSIIKGLAKGLAEHF) and the bombinin H-type peptide (H-BO1) (IIGPVLGLVGKALGGLL). The prediction of secondary structures showed that both BO1 and H-BO1 peptides adopted α-helical structures, exhibited broad-spectrum antimicrobial effect against Gram-positive and Gram-negative bacteria and yeast as well as anticancer effects against human hepatoma cell lines tested (Hep G2/SK-HEP-1/Huh7). Therefore, these peptides may represent novel therapeutic strategies for liver diseases [152].

## 4. Conclusions

AMPs/ACPs provide new strategies for cancer treatment due to their efficacy against tumor cells obtained at low concentrations without toxicity to normal tissues. Many limitations in the use of peptides, such as poor stability, proteolytic degradation, potential toxicity, low bioavailability, specific delivery to tumor cell, have been overcome by the chemical modifications including amino acids substitution, nanoparticles loading, fusion with cell penetrating peptides.

The next goal is to improve the transfer of natural or engineered AMPs/ACPs into the clinic. A large number of AMPs are already in the pre-clinical stage and several are under evaluation in clinical trial as antibacterial but only few as anticancer peptides (Table 2). AMPs and ACPs are the only class of compounds that might effective against polymicrobial co-infections as well as cancer.

The improvement in chemical synthesis strategies of AMPs/ACPs and the study of their structures could clarify the structure-activity relationship thus allowing to enhance the activity and reduce the negative side effects in infectious disease and cancer treatment.

## Figures and Tables

**Table 1:** List of the most representative antimicrobial peptides acting as anticancer molecules.

AMP Name	Amino Acid Sequence	Structure Class	Net Charge	Source	Tumor Target	Mechanism	Ref.
Cathelicidins LL37 hCAP18	LLGDFFRKSKEKIGKEFKRIVQRIKDFLRNLVPRTES	unknown	6	Human	HTC/STC	MP/Apoptosis	[84,85,86,87,88]
α-Defensin-1 HNP-1	ACYCRIPACIAGERRYGTCIYQGRLWAFCC	beta	3	Human	HTC/STC	Apoptosis	[89,90]
Human b-defensin-3 (hBD3)	GIINTLQKYYCRVRGGRCAVLSCLPKEEQIGKCSTRGRKCCRRKK	mixed structure	11	Human	HTC/STC	MP	[91]
Lactoferricin B(LfcinB)	FKCRRWQWRMKKLG APSITCVRRAF	beta	8	Bovine	HTC/STC	MP/Apoptosis	[92,93]
Gomesin	* ZCRRLCYKQRCVTYCRGR	beta	6	Spider	STC	MP	[94]
Mastoparan-C (MP-C)	LNLKALLAVAKKIL	helix	4	Venom	STC	Apoptosis	[95]
Cecropin B	KWKVFKKIEKMGRNIRNGIVKAGPAIAVLGEAKAL	unknown	8	Silk moth	HTC/STC	MP/Apoptosis	[96,97]
Magainin 2	GIGKFLHSAKKFGKAFVGEIMNS	helix	3	Frog	HTC/STC	MP	[98,99]
CA-MA-2	KWKLFKKIPKFLHSAKKF	helix	8	Hybrid	STC	MP	[100]
BuforinIIb	RAGLQFPVG[RLLR]3	unstructured	7	Frog	HTC/STC	Apoptosis	[101]
Brevenin-2R	KFALGKVNAKLQSLNAKSLKQSGCC	helix	5	Frog	STC	LDP	[102]
LFB	GLFSVVKGVLKGVGKNVSGSLLDQLKCKISGGC	unknown	4	Frog	STC	MP	[103]
Phylloseptin-PHa	FLSLIPAAISAVSALANHF	helix	2	frog	STC	MP	[104]
Ranatuerin-2PLx	GIMDTVKNAAKNLAGQLLDKLKCSITAC	helix	2	frog	STC	Apoptosis	[105]
Dermaseptin-PS1	ALWKTMLKKLGTVALHAGKAALGAVADTISQ	helix	5	frog	STC/ ICD	MP	[106,107]
Dermaseptin (DPT9)	GLWSKIKDAAKTAGKAALGFVNEMV	helix	2	*Phyllomedusatarsius*	STC	MP	[108]
chrysophsin-1	FFGWLIKGAIHAGKAIHGLI	helix	6	Red sea bream	HTC/STC	MP	[109]
Ss-arasin	SPRVRRRYGRPFGGRPFVGGQFGGRPGCVCIRSPCPCANYG	bridge	8	Indian mud crab	STC	uncharacterized	[110]
**Turgencin A and B**	**GIKEMLCNMACAQTVCKKSGGPLCDTCQAACKALG**	**helix**	**3**	***Synoicum turgens***	**STC**	**uncharacterized**	**[111]**
D-K6L9	LKLLKKLLKKLLKLL	helix	3	Engineered	STC	MP	[112]
KLA	RRQRRTSKLMKRGGKLAKLAKKLAKLAK(KLAKLAK)2	unknown	19	Engineered	STC	MP	[113]
LTX-315	K-K-W-W-K-K-W-Dip-K	unknown	5	Engineered	HTC/STC	MP/ICD	[114,115]
TAT-RasGAP317-326	RRRQRRKKRGGGDTRLNTVWMW	unknown	8	Engineered	STC	MP	[116]

* Z, pyroglutamic acid; HTC, hematological tumor cells; STC, solid tumor cells; MP, membrane permeabilization; LDP, lysosomal death pathway; ICD, immunological cell death.

**Table 2:** Antimicrobial peptides under evaluation in clinical trial of cancer diseases.

Peptide Sequence	Clinical Stages	Indications	Identifier Number
LL37: LLGDFFRKSKEKIGKEFFJVQRIKDFLRNLVPRTES	Phase II	Melanoma	NCT02225366
LTX315: KKWWKK-Dip-K-NH_2_	Phase I Phase I Phase II	Solid tumors	NCT01058616 NCT01986426 NCT03725605
SGX942 Dusquetide:RIVPA	Phase III	Head and neck cancer	NCT03237325

**Table 3:** List of the most representative antimicrobial peptides acting against HCC.

AMP Name	Amino Acid Sequence	Structure Class	Net Charge	Source	Mechanism	Ref.
Tv1	TRICCGCYWNGSKDVCSQSCC	mixed	2	Venom	Apoptosis	[142,143,144]
SALL4 peptide FFW	RRKFAKFQWI	mixed	4	Engineered	Silencing tumor-suppressor gene	[145]
SP94	SFSIIHTPILPL	unknown	0	Human	Apoptosis	[68,146]
R-Tf-D-LP4	KWTWKNSNGATWALNVATELKKEWTWSHRPYIAH	unknown	5	Human	Apoptosis	[147]
GG-8-6	cyclo-VLPILLVL	cyclic	0	Plant	Apoptosis	[148]
β3	DLYYLMDLSYSMKGGDLYYL MDLSYSMKGGDLYYLMDLSYSMK	unknown	3	Engineered	Anti adhesion activity	[149]
CecropinX	RWKIFKKIEKMGRNIRDGIVKAGPAIEVLGSAKAIGK	unkonown	10	Silk moth	Apoptosis	[150]
GW-H1	GYNYAKKLANLAKKPANALW	helix	4	Engineered	Apoptosis	[151]
Bombinin	GIGSAILSAGKSIIKGLAKGLAEHF IIGPVLGLVGKALGGLL	helix unkonown	31	Fire-bellied toad	Apoptosis	[152]
